# Reply to Wallace et al. Comment on “Panuccio et al. Quality of Assessment Tools for Aphasia: A Systematic Review. *Brain Sci.* 2025, *15*, 271”

**DOI:** 10.3390/brainsci15111234

**Published:** 2025-11-17

**Authors:** Francescaroberta Panuccio, Giulia Rossi, Anita Di Nuzzo, Ilaria Ruotolo, Giada Cianfriglia, Rachele Simeon, Giovanni Sellitto, Anna Berardi, Giovanni Galeoto

**Affiliations:** 1 Department of Human Neurosciences, Sapienza University of Rome, Viale dell’Università, 30, 00185 Rome, Italy; francescaroberta.panuccio@uniroma1.it (F.P.); giu.rossi@uniroma1.it (G.R.); anitadinuzzoe@gmail.com (A.D.N.); ilaria.ruotolo@uniroma1.it (I.R.); giada.cianfriglia@gmail.com (G.C.); giovanni.sellitto@uniroma1.it (G.S.); anna.berardi@uniroma1.it (A.B.); 2 Department of Public Sciences and Infectious Diseases, Sapienza University of Rome, 00185 Rome, Italy; 3 Department of Neuroscience, Rehabilitation, Ophthalmology, Genetics and Maternal Child Health (DINOGMI), University of Genoa, 16126 Genoa, Italy; rachele.simeon@edu.unige.it; 4 IRCCS Neuromed, Via Atinense, 18, 86077 Pozzilli, Italy

We would like to thank the authors of the commentary for the attention given to our work and for the observations provided, which we believe may contribute to improving the manuscript [1].

Our review included a considerable number of articles (*n* = 238), with 164 assessment tools identified. A detailed analysis of the issues raised allowed us to identify some discrepancies caused by formatting errors, given the large amount of data to be managed in the processes of classification and evaluation reported in the tables. These discrepancies will be corrected through a systematic re-evaluation of each included study, with the aim of ensuring greater methodological consistency and transparency in the results. These mainly concerned the incorrect allocation of COSMIN checklist items and minor inaccuracies in the citation of some studies. For example, we inadvertently inverted the following two rows: “*The structural validity of the original English-language SAQOL-39 [2] and SAQOL-39g [3] is rated as insufficient despite both studies reporting results of Exploratory Factor Analysis, while the Japanese SAQOL-39 [4] received a positive rating despite no reported factor analysis at all in the cited article*”. While we understand the observations concerning the documentation of quality ratings reported in Table 3, it is important to note that our goal was to map the existing literature, not to produce a definitive assessment of each tool’s methodological quality. From this perspective, our work serves as a useful foundation for future reviews that may focus more specifically on detailed quality evaluation.

We also acknowledge the observations regarding the use of COSMIN and the categorization of instruments. In particular, with respect to the reference to Mokkink [5], we clarify that the authors actually adopted the updated 2020 version and that the incorrect citation was a clerical error. Citation and attribution errors will also undergo thorough revision in order to ensure rigorous alignment between the sources and evaluations.

We further recognize that in some included studies, such as Koenig-Bruhin et al. (2016) [6], the proportion of participants with aphasia was not specified. This certainly represents a limitation in terms of generalizability; however, the decision to include these studies was motivated by the fact that they reported psychometric data relevant to the assessment tools. These studies were, nevertheless, considered with caution, acknowledging the implications for applicability to the target population.

The aim of this review was to identify currently available measurement tools (such as scales, tests, and questionnaires) that assess various aspects of communication and cognition in individuals with aphasia and in which psychometric properties are documented in scientific papers and manuals. The purpose was to provide a comprehensive overview of existing instruments rather than an exhaustive quality appraisal.

Regarding the exclusion of manuals from the inclusion criteria, we reiterate that our systematic review was designed to include only peer-reviewed articles published in indexed scientific journals. This methodological choice, in addition to ensuring rigor in the selection process, was also intended to guarantee equitable access to the available evidence: manuals are often subject to economic barriers that limit their consultation in clinical practice. We believe that the availability of data from sources accessible to all clinicians and researchers represents an essential requirement to promote the transferability of evidence and equity in care.

In conclusion, we confirm the authors’ commitment to correcting the discrepancies identified, while at the same time maintaining the methodological choice of privileging exclusively peer-reviewed sources, in line with criteria of rigor, transparency, and accessibility.
